# Malignant Peripheral Nerve Sheath Tumors in Africa: A Clinicopathological Study

**DOI:** 10.5402/2011/526454

**Published:** 2011-04-11

**Authors:** Peter M. Nthumba, Paul Irungu Juma

**Affiliations:** Plastic, Reconstructive and Hand Surgery Unit, AIC Kijabe Hospital, Kijabe 00220, Kenya

## Abstract

*Introduction*. Malignant peripheral nerve sheath tumors (MPNSTs) are rare, aggressive soft tissue sarcomas associated with poor prognosis, that most commonly affect patients aged 20 to 50 years, but have also been reported in children. There is little reported in literature on these tumors in Africa. *Materials and Methods*. A search of the hospital pathology database between 1992 and 2008 revealed 333 nerve sheath tumors, of which 31 were MPNSTs. Four representative case reports are presented. *Discussion*. MNPSTs have rarely been reported from sub-Saharan Africa; in this study, they constituted 9.3% of all nerve sheath tumors. The trunk (42%) and limbs (45%) were the most frequently affected anatomical sites. Late presentation of malignant lesions in this environment is exemplified by the four case presentations patients. *Conclusions*. This report confirms observations from studies on MPNSTs from other environments. Anatomically centrally located MPNSTs may have a higher incidence in sub-Saharan Africa than in the West. Because NF1-associated MPNSTs are difficult to diagnose clinically, and because surgery is the only mode of therapy that offers a complete cure, a lifetime follow-up is important, as this would enable diagnosis of early lesions amenable to surgical extirpation.

## 1. Introduction

Benign and malignant peripheral nerve sheath tumors may occur sporadically or in conjunction with neurofibromatosis type 1 [1]. Malignant peripheral nerve sheath tumors (MPNSTs) are rare, aggressive soft tissue sarcomas that either arise from a peripheral nerve or show nerve sheath differentiation. Up to 11% may arise secondary to radiation therapy, with latencies of 20 to 40 years [2]. MPNSTs account for 10% of all soft tissue sarcomas and are associated with a high risk of metastases and local recurrence, resulting in poor prognosis. They metastasize to the lungs, liver, brain, bone, regional lymph nodes, and skin, amongst other organs [1, 3]. MPNSTs most commonly affect patients aged 20 to 50 years, but have also been reported in children [4–6]. 

Approximately 50% of MPNST occur in patients with neurofibromatosis type 1 [7]. MPNSTs reduce the life expectancy of NF1 patients by up to 15 years. Patients with Nf1 have an 8% to 13% lifetime risk of developing MPNST [8, 9]. 

Factors associated with the development of MPNSTs include NF1 patients with many, extensive, or centrally located plexiform neurofibromas, as are nodular plexiform tumors associated with large peripheral nerve sheaths and extensive tumors involving the brachial, lumbar, or sacral plexus. Microdeletions of the NF1 locus may also be indicators of high risk for MPNST development [3, 10]. Most NF1-associated MPNSTs arise within preexisting plexiform neurofibromas; patients with Nf1 therefore should be under lifetime surveillance for the development of MPNST [10]. Rhabdomyosarcomas have also been reported to occur in NF1 patients [11]. 

There is little reported in the literature on malignant peripheral nerve sheath tumors in Africa [12–15]. Although rare, some reports from the USA suggest a higher incidence amongst Blacks than Caucasians [16]. There are no comparative data from sub-Saharan Africa. The authors sought to find the demographics of MPNSTs amongst sub-Saharan Africans.

## 2. Materials and Methods

Records on surgical specimens submitted for histological examination to the hospital pathology department between 1992 and 2008 reported as peripheral nerve sheath tumors were retrieved from the database. Clinical records of four patients who presented to the authors' hospital and were found to have malignant peripheral nerve sheath tumors were reviewed and are reported; these were not part of the data from the pathology department. An Internet/PubMed search of sub-Saharan published literature on MPNSTs was made.

## 3. Results

There were a total of 333 peripheral nerve sheath tumors diagnosed over the 16 years; 31 patients (15 females and 16 males) had a histological diagnosis of malignant peripheral nerve sheath tumor (Table 1). Patients with MPNSTs had a mean age of 34 years (8 to 80). The trunk, upper limbs, lower limbs, and head and neck constituted 42%, 22.5%, 22.5%, and 10%, respectively. In one patient (3%), the anatomic site of origin was not indicated. Fifteen of the 31 patients (48%) had neurofibromatosis type 1 (NF1). Sixty percent of female patients had NF1, while 40% of males had NF1 (Table 1). 

The authors found only four papers from sub-Saharan Africa that had reported MPNSTs [12–15]. 

## 4. Case Presentations

**Case Report 1**

**Figure 1:**
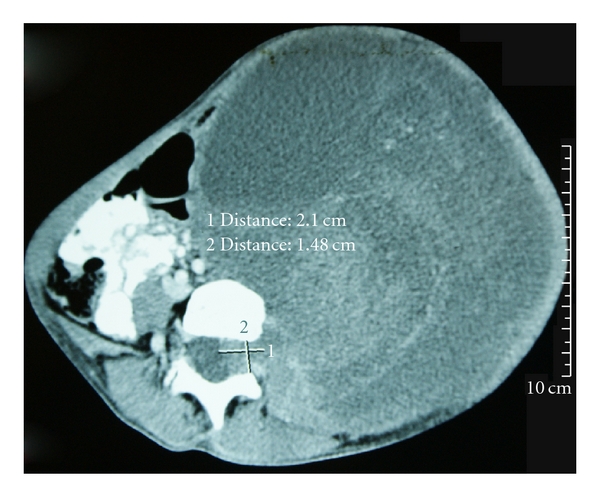
Coronal CTScan showing large retroperitoneal tumor extending and abutting the spinal cord. Abdominal contents are compressed paracolic gutter.

A 21-year-old female patient with NF1 presented with lower limb pain and weakness for one year. She also had a large abdominal mass associated with early satiety and constipation for four months prior to presentation. She was cachectic, with a weight of 42 kg. A CT Scan showed an extensive retroperitoneal tumor compressing the diaphragm cranially, and filling the pelvic cavity, likely of paraspinal origin (Figure 1). At surgery, a large fairly well-encapsulated retroperitoneal tumor involving the nerve roots of L3, 4, and 5 was excised. The vertebral bodies of L4 and L5 had been eroded, and an iliac crest bone strut was used to support the L4 vertebral body. The tumor weighed 5.5 kg. Histology was reported as MPNST. Postoperatively, the patient's symptoms improved; she was able to eat well and ambulate.

**Case Report 2**

**Figure 2:**
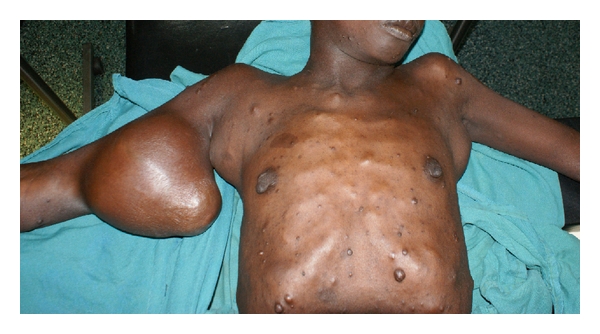
Pre-operative picture of case report 2: note bilateral axillary tumors in addition to large mass over right arm.

**Figure 3:**
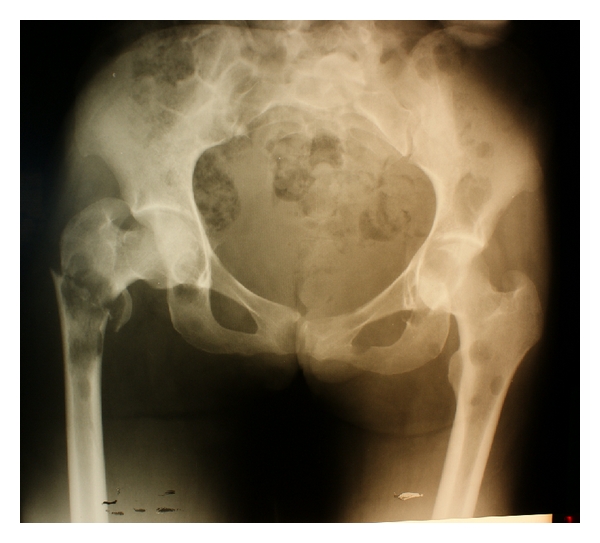
Pathological fracture of the left hip, with contralateral osteolytic lesions.

A 19-year-old female with NF1 presented with a one-week history of left thigh pain and inability to walk. She had multiple large bilateral upper limb masses (bilateral axillary and right arm) that were associated with pain and paraesthesiae (Figure 2). A skeletal survey (whole body radiographs) revealed a pathological fracture of the neck of left femur (Figure 3), as well as extensive bone lesions involving the right femur, both humeri and lumbar vertebrae. The three large masses were excised, with histopathological results revealing a malignant MPNST in the right forearm tumor and severe dysplasia in the right axillary mass. The left axillary mass was a benign nerve sheath tumor.

The femur fracture was fixed; biopsies revealed metastatic MPNST. Subsequently, while still in the hospital, she developed bilateral lower limb weakness and stumbled and fell while walking. She sustained pathological fractures of the 4th and 5th lumbar vertebra and the right humerus. After operative fixation of the humeral fracture, she was referred elsewhere for chemoirradiation and got lost to followup.

**Case Report 3**

**Figure 4:**
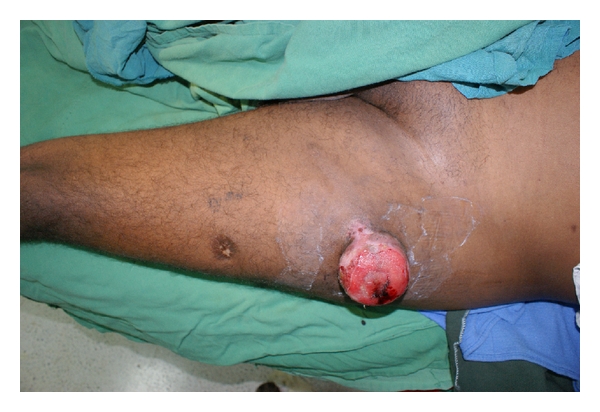
Preoperative picture of third case report: solitary malignant peripheral nerve sheath tumor.

A 35-year-old male presented with an ulcerated, pedunculated mass on the left thigh for a period of 5 years (Figure 4). He had no stigmata of NF1. After a biopsy showing it to be a malignant peripheral nerve sheath tumor, a wide excision was performed; negative margins were confirmed at histology.

**Case Report 4**

**Figure 5:**
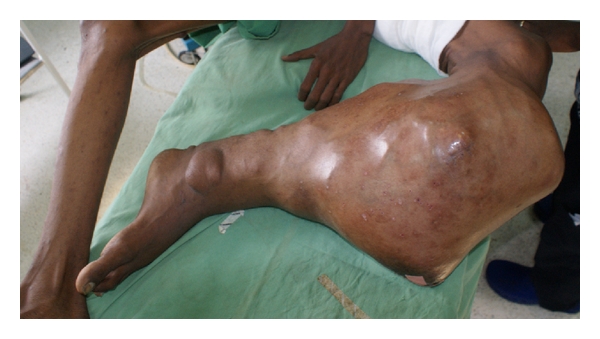
Malignant peripheral nerve sheath tumor of the left leg.

**Figure 6:**
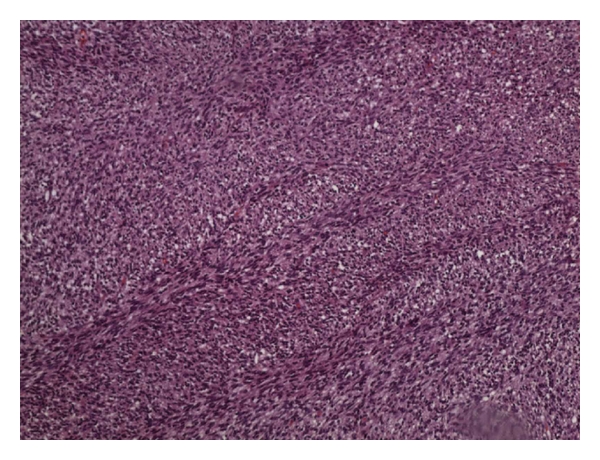
Photomicrograph showing herringbone pattern of malignant spindle cell tumor with mitosis (arrows) (hematoxylin and eosin stained; magnification ×100).

**Figure 7:**
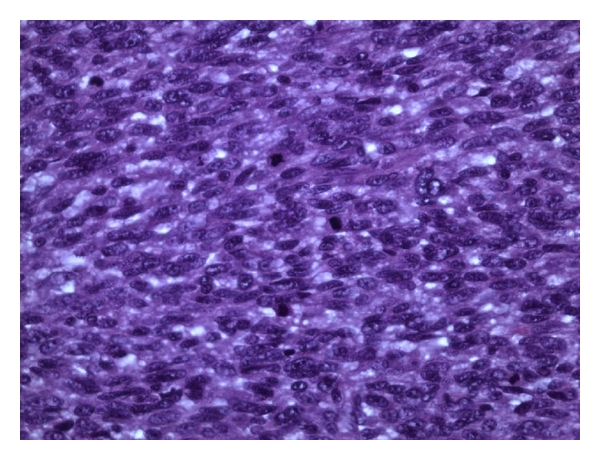
High-magnification photomicrograph showing malignant spindle cell tumor with frequent mitosis (arrows). Note high cellularity and nuclear polymorphism (hematoxylin and eosin stained; magnification ×400).

A 21-year-old male with NF1 presented with a large painful, ulcerated mass involving the proximal two-thirds of the left leg (Figure 5). He had had the mass for many years, but had noted a rapid increase in the size of the tumor in the five months prior to presentation. A clinical diagnosis of MPNST was made. He had no evidence of metastases, and an above-knee amputation was performed. Histological examination revealed MPNST (Figures 6 and 7).

The patient was ambulant with crutches 24 hours after surgery, with minimal pain.

## 5. Discussion

Late presentation of patients with advanced malignancies is not uncommon in sub-Saharan Africa. Of the four representative patients, only one had a surgically curable lesion, even though he presented late with an ulcerated lesion; a second potentially curable lesion was the fourth patient who underwent an above-knee amputation. One of the other two patients had extensive bony lesions, resulting in a number of pathological fractures: femoral, humeral fracture, and lumbar vertebral fractures. The quality of life of the first and third patients improved significantly postoperatively. 

The rarity of MPNSTs in the sub-Saharan population is attested to by the few reports and low numbers from the region [12–15]. Odebode et al. in a clinicopathological review covering 22 years, found 98 patients with nerve sheath tumors, of whom 3.1% had PMNSTs [13]. In Samaila and Adewuyi's review of 382 cutaneous malignancies, only three were MPNSTs, representing 0.8% of the tumors [14]. Two large studies from Kenya, one on neural tumors of the head and neck, and another on sarcomas of the head and neck, did not find any MPNSTs [17, 18], while Tenge et al., in establishing a cancer registry of Western Kenya, did not report any MPNSTs from a patient population of over 5000 with different types of cancers [19]. Adeyemi et al. found MPNSTs in 6.5% of all head and neck cancers in a Nigerian institution—four of the five patients were females [15]. 

In the current study, 9.3% of histologically diagnosed nerve sheath tumors were MPNST. One patient had a triton tumor; MPNSTs with rhabdomyoblastic differentiation may have a more aggressive clinical course than other MPNSTs [20]. Sixty-one percent of patients were aged between 20 and 40 years, consistent with the reports of other authors [4], while 15 (48%) of patients in the current study had NF1, similar to other studies [7].

MPNSTs in other studies most commonly involve major nerve trunks, such as the brachial plexus, sacral plexus, or the sciatic nerve and often present with pain and motor or sensory deficits [1, 21]. However, though pain, neurological deficits, and growing masses are the main clinical indicators of malignancy, they are also presenting symptoms in some patients with benign plexiform neurofibromas, making it difficult to differentiate on clinical grounds only, between malignant and benign lesions [7, 10, 22, 23]. In the current study, the trunk was the commonest involved anatomic site (42%), a finding reported in some studies [24]. 

The MPNSTs in multiple bones in one patient were either metachronous tumors or metastatic from the upper extremity lesions. Pathological fractures from MPNSTs are very rare but have been reported [25]. Sar and Eralp proposed an aggressive therapy for spinal metastatic disease [25]. In the current paper, the patient who sustained lumbar vertebral pathological fractures/collapse, after stabilization of the femoral and humeral fractures, was referred for oncologic care. 

Patients with MPNSTs may have a poorer prognosis than those with sporadic tumors, because NF1-associated MPNSTs tend to be centrally located, and therefore less amenable to surgery [7]. Poor prognostic factors include tumor size (greater than 5 cm), local invasion, and histologic grade. Good prognostic factors include duration of less than 6 months, clear surgical margins, and an age less than 30 years [26]. 

Aggressive wide resection (at least 10 cm margins), including limb amputation, is advocated for MPNST, as in the fourth case report. While this may be impossible in anatomically centrally located sites, unfortunately, surgery is the only modality of therapy that can achieve cure [22, 27, 28]. Limb-sparing excision with chemoradiotherapy in combination or separately is increasingly favored. The role of chemotherapy for MPNSTs is not yet defined, although a favorable response in both children and adults has been reported [1, 7, 12, 22]. Local recurrence of MPNST is high, even with multimodal therapy, reported to be between 30% to 65% [27, 28]. 

While patients with NF1-associated MPNSTs presented at an average age of 27 years, those with sporadic MPNSTs had a mean age of 42 years, a finding consistent with other reports that have shown NF1-associated MPNSTs present at an earlier age [7, 23].

 Retroperitoneal MPNSTs have a very poor prognosis, because of an insidious onset, local invasiveness, and frequently late diagnosis. Further, surgical excision is incomplete, and chemotherapy and radiotherapy are reportedly ineffective in retroperitoneal MPNSTs [24]. Patients often succumb to systemic metastatic disease [29]. Surgical excision of the giant retroperitoneal peritoneal tumor in the patient reported here was of immediate benefit, improving her ability to eat and retain food, as well as ambulate, as was the leg amputation for the fourth patient. 

There was no difference in the incidence of centrally located tumors between NF1-associated and sporadically occurring MPNSTs in the current study. This high incidence of nonextremity lesions in sub-Saharan Africa MPNSTs may be equated with poor prognosis, resulting from incomplete resections, recurrences, and deaths, especially in an environment where other modalities of therapy are either unavailable or unaffordable.

The final outcomes of the 31 patients reported here are unknown. Of the four representative case reports, two returned for followup; the surgical margins in both patients were considered adequate, and adjuvant therapy was considered unnecessary. The outcome of the two female patients remains unknown as they did not return after oncology referrals. Because negative surgical margins were not achievable in the two patients and because of extensive metastases in one, it is likely that they may have succumbed to the disease and died. Oncologic services in Kenya remain either difficult to access because of large volumes of patients awaiting therapy in public institutions or inaccessible because of cost in private hospitals.

## 6. Conclusions

Malignant peripheral nerve sheath tumors are rare malignancies that have been infrequently reported from sub-Saharan Africa. This clinicopathological report confirms observations from studies on MPNSTs from other environments: they are rare and 48% occurred in NF1 patients, in whom they occur at a younger mean age (27 years), in comparison to patients with solitary MPNSTs (42 years). 

Unlike most Western studies, however, there was no difference in the incidence of centrally located MPNSTs (which have a poor prognosis), between NF1-associated MPNSTs and solitary MPNSTs. 

Because malignant lesions in NF1 patients are difficult to diagnose clinically and because surgery is the only mode of therapy that offers a complete cure, a lifetime followup is important, as this would enable diagnosis of early lesions amenable to surgical extirpation. 

## Figures and Tables

**Table 1:** Characteristics of patients with MPNST.

Sex	Age (yrs)	NF1 status	Anatomic site
F	22	NF1	Site Unknown
F	24	NF1	Retroperitoneum
M	29	NF1	Neck
M	36	Solitary	Leg
M	38	Solitary	Axilla
F	64	Solitary	Thigh
F	18	Solitary	Forearm
M	?	Solitary	Arm
F	26	NF1	Chest wall
M	29	Solitary	Thigh
M	30	NF1	Leg
M	18	Solitary	Hand
M	22	NF1	Back
F	30	NF1	Neck
M	22	NF1	Back
M	38	Solitary	Axilla
F	38	Solitary	Leg
F	26	NF1	Chest wall
M	70	Solitary	Back
F	80	NF1	Back
F	25	Solitary	Leg
M	12	Solitary	Back
F	36	Solitary	Breast
M	43	NF1	Scalp
F	78	Solitary	Neck
M	10	NF1	Arm
F	8	NF1	Gluteal
M	27	Solitary	Back
M	70	Solitary	Retroperitoneum
F	25	NF1	Hand
F	29	NF1	Anterior abdominal wall

## Abbreviations

MPNST(s): Malignant peripheral nerve sheath tumor(s)
NF1: Neurofibromatosis type 1
CTScan: Computerized tomography scan
USA: United States of America.
